# On the job training in the dissection room: from physical therapy graduates to junior anatomy instructors

**DOI:** 10.1186/s12909-022-03390-y

**Published:** 2022-05-10

**Authors:** Smadar Peleg, Tomer Yona, Yuval Almog, Alon Barash, Ruth Pelleg-Kallevag

**Affiliations:** 1 Department of Physical Therapy, Zefat Academic College, Zefat, Israel; 2 School of Human Movement and Sport Sciences, Levinsky-Wingate Academic Center (Wingate Campus), Nethania, 4290200 Israel; 3 Department of Biomedical Engineering, Technion - Israel Institute of Technology, Haifa, Israel; 4 Faculty of Medicine and Health, University of Sydney, Camperdown/Darlington, NSW Australia; 5 The Azrieli Faculty of Medicine, Bar Ilan University, 8 Henrietta Szold St, Safed, Israel; 6 Department of Anatomy and Anthropology, Tel Aviv University, Tel Aviv-Yafo, Israel

**Keywords:** Anatomy education, Physical therapy education, Near-peer teaching, Lewinian experiential model

## Abstract

**Background:**

The training of near-peer (NP) teachers and junior faculty instructors received major attention as a possible solution for the shortage of experienced anatomy instructors in faculties of medicine and health professions. Several studies described the training of NP teachers and junior instructors (≤ 2 years of teaching experience) using various methods. However, few publications include On the Job Training (OJT), which enables reflection and performance evaluation and encourages professionals to cope with their blind spots. Previous publications describing OJT did not include formal observation of the NP teacher or junior instructor. Therefore, this study aimed to present a novel approach to OJT inclusion during prosection laboratories based on the Lewinian experiential model.

**Methods:**

Eight physical therapy (PT) graduates were recruited as junior anatomy instructors into the prosection laboratories. All participated in a unique training program during two consecutive academic years (2017, 2018) and received OJT during the teaching sessions. Two questionnaires were filled out to evaluate the educational impact of the training program. Eighty-three first-year PT students participated in prosection laboratories in anatomy taught by junior instructors, and filled out a questionnaire evaluating the performance of both junior and senior instructors. In addition, we compared the final grades in anatomy obtained by students taught by senior instructors to the grades of those taught by junior instructors.

**Results:**

Each junior anatomy instructor participated in four OJT sessions. Based on self-reported measures, all professional and didactic aspects of the training program received a median score of 4.5 or higher on a five-point Likert scale. Students obtained similar grades in anatomy when taught by junior instructors compared with senior ones, and were similarly satisfied from the teaching performance of both senior and junior anatomy instructors.

**Conclusions:**

OJT is applicable in a small-sized PT program facing a shortage of anatomy instructors. Including junior anatomy instructors in prosection laboratories for PT students is a viable solution to the shortage of experienced anatomy instructors. Further study, involving a larger cohort with a longer follow up will strengthen the preliminary results presented here.

**Supplementary Information:**

The online version contains supplementary material available at 10.1186/s12909-022-03390-y.

## Background

Gross anatomy courses are considered essential to the education of medical and health professions students [1–5]. Over the past decades, pedagogical concepts of “authentic learning” were adapted to gross anatomy courses, whereby clinical knowledge was integrated into introductory anatomy courses via active learning [6–10].

The integration of problem-based learning and the inclusion of computer-based learning, medical imaging and ultrasound led to a reduction in the number of frontal lectures and dissection laboratory hours and the transition to a multi-modal learning content [1, 11–19]. In addition, reduced laboratory hours and limited availability of skilled dissectors were mentioned as possible risks to decreased knowledge in anatomy [18, 20, 21]. Furthermore, a shortage of cadavers, adequate dissection facilities, and experienced anatomy instructors challenge the inclusion of dissection/prosection laboratories in anatomy syllabi [22–24]. Nevertheless, even scaled-down dissection or prosection laboratories are considered a fundamental part of all anatomy courses [1, 4, 5, 25–27]. In many cases, dissection laboratories were replaced with prosected material, and a shift towards peer and near-peer (NP) teaching, whereby students are taught by students from senior years, has been proposed as a viable solution, enabling NP teachers (NPT) to develop their teaching skills before becoming residents [27–37].

The incorporation of junior faculty into the faculty of medicine after being trained in anatomical science and teaching obligations is another strategy [38, 39]. Thus, a solution to shortage of experienced anatomy instructors is including NPT or junior faculty in the dissection/prosection laboratories [39, 40]. Appropriate training, emphasizing pedagogic, didactic and professional aspects in anatomy instruction, is essential in both cases [39–44].

Several authors described training of NPT and junior faculty, incorporating various methods, such as the microteaching method that offers simulations of teaching and reflection on performance, modules of teaching skills, ongoing peer evaluation, and performing a prosection under supervision [44, 45]. Others included training in gross anatomy and neurosciences, and practicum experiences as part of their faculty training [39]. All programs were regarded as beneficial and received high ratings by students, NPT and junior faculty [39, 41, 44, 45].

As gaining anatomical skills is more straightforward than gaining didactic communication skills and proficiency, a formal peer observation process and follow-up programs were recommended to support the NPT or junior faculty in their first steps [41, 44, 46, 47]. On the job training (OJT) is an excellent method for this purpose, allowing fast and efficient changes in the goals of course syllabi and teacher/instructor roles in light of the challenges described above [48, 49].

Initially developed in the field of Economics, OJT has a central role in lifelong learning in medicine and health professions, however the majority focus on describing policies rather than describing the process in depth [50–57]. Interventions on practicing psychomotor skills of surgeons, improving evidence based practice for clinicians and clinical scientists and improving communication skills of residents, emphasize the importance of a structured mentoring process with high availability of the senior instructor to the trainees [54, 55, 57].

Strategies incorporated into OJT include reflection that reveals gaps in knowledge, skills or attitudes, and performance evaluation, which is tailored to encourage professionals to cope with their blind spots [58–62].

Previous publications describing OJT in anatomy education included mentoring, debriefing and providing feedback to the trainees [32, 36, 40]. Most did not include formal observation of the trainee, except Evans and Cuffe, who described an informal observation process. However, they did not describe the process in depth [46].

A key element of the Lewinian experiential model is formal observation of the trainee, followed by structured reflection and feedback [63, 64]. This has not been described previously. Therefore, a novel approach to OJT inclusion during the prosection laboratories, adapted to PT clinical content, is presented here. In this article, we describe a unique training program for junior anatomy instructors (≤ 2 years of teaching experience) tailored to the needs of an undergraduate PT department, established in 2010 in Zefat Academic College (Zefat, Israel). Due to a shortage of skilled anatomy instructors and limited resources, junior instructors were incorporated into the prosection laboratories.

The objectives of the present study were: to describe a new program of training junior anatomy instructors for prosection laboratories, adapted to the needs of PT curricula, to evaluate the training programs’ educational impact, to evaluate junior anatomy instructors’ performance, and finally, to evaluate the academic achievements of PT students taught by the junior anatomy instructors.

## Materials and methods

### Junior anatomy instructors

Following the approval by Zefat Academic College’s Ethics committee (no. 07/2017) eight PT graduates (six males and two females), between the ages of 24 and 28, participated in a unique training program during two consecutive academic years (2017, 2018). The junior anatomy instructors’ background in anatomy was the gross anatomy course taken during their first academic year in the PT department. Additionally, all served as tutors during their second year and assisted with preparing the prosection laboratories during their third or fourth academic years before participating in the training program. Therefore, their teaching experience was less than two years.

### Training program

Based on previous publications [13, 25, 39, 41–44], a training program was developed to meet the needs of the PT Department at Zefat Academic College.

and implemented during 2017 and 2018.

The objectives of the training program were threefold: (1) to expand the junior instructors' knowledge in anatomy; (2) to provide the junior instructors with didactical and pedagogical skills; and (3) to improve the junior instructors' performance through providing both a supportive environment and continuous feedback. Table 1 includes the expanded learning goals of the training program.

**Table 1:** Learning goals of the training program (Workshop and On the Job Training)

Learning goal ^a^	Expanded learning goals based on the Lewinian experiential learning model ^b^
The tutor can perceive group processes and positively influence it	Presenting the topics of a session to help retain structured instruction (maintaining focus)
The tutor demands active knowledge from the students using active teaching modules	Giving a simple and accurate explanation
	Giving a short instruction (≤ 15 min) followed by structured active learning of the students
The tutor can explain the defined clinical context to each regional topographical course	Providing a short clinical context when relevant
	Combining the use of a body and a preparate^c^ in order to complete the three-dimensional and multilayer explanation
	Situation awareness (SA)^d^

^a^ Based on Shiozawa et al., 2010 [41, 45]

^b^ Based on Kolb 1984 [63]

^c^ Preparate – a prosected body part

^d^Learning goal developed by the authors based on Wolff et al., (2020) [65] and Harden and Laidlaw (2017) [8, 9, 66]

**Fig. 1:**
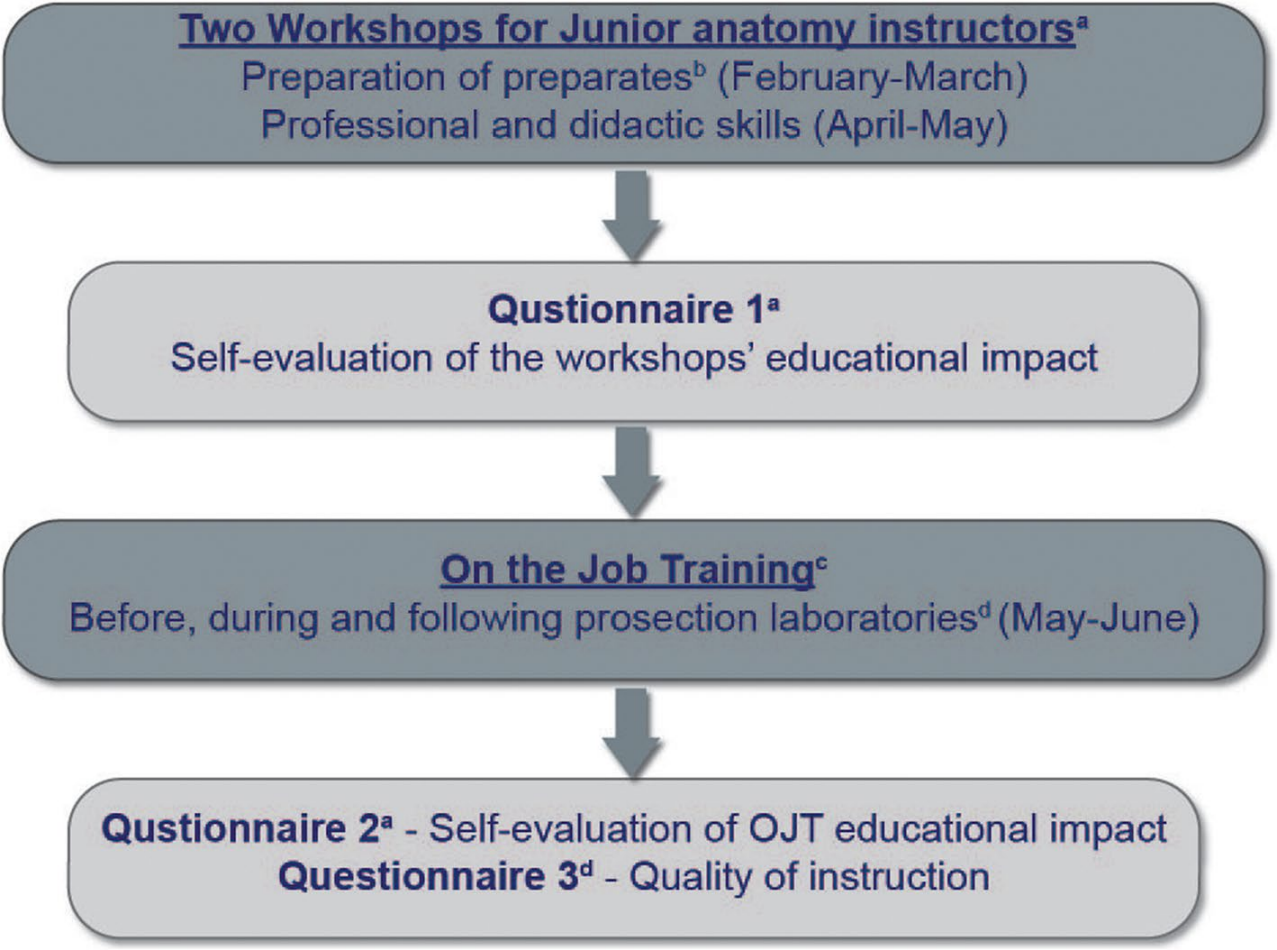
Flow chart of the training program and methodology. ^a^Two workshops for junior anatomy instructors,2 years of teaching experience, *n* = 8, 32 h each. ^b^A prosected body part. ^c^OJT supervised by a senior instructor (≥ 10 years of teaching experience). ^d^Prosection laboratories for first-year students during 2017–2018 academic years (*n* = 85)

The training program consisted of two workshops: the first workshop focused on prosection preparation, the second workshop was dedicated to principles in anatomy instruction (32 h each). Following the two workshops, OJT was carried out, whereby the junior instructor received feedback from a senior anatomy instructor prior to, during, and following each teaching session. Self-evaluation of the workshops and OJT was carried out following the workshops and OJT (see Fig. 1 for a flow chart of the methodology).

#### The workshop on preparing prosections

This workshop was led by two senior instructors (SP and RPK, ≥ 10 years of teaching experience) and was conducted during the semester break of each year (February–March). A didactical lecture on ethics in the dissection room was delivered during the first session, followed by demonstrations of cutting techniques. The main aspects were: working with the aid of an atlas, working from distal to proximal, working slowly while exposing neurovascular structures, working with scissors vs. working with a Stanley knife based on the size and depth of the structure.

Each junior anatomy instructor was assigned to a specific region, e.g., upper arm, forearm, intrinsic foot muscles, knee joint and ligaments etc. Two senior instructors inspected all prosected materials (SP and RPK) to ensure high quality, e.g., separation between muscles and layers, exposing neurovascular structures, identification of the origin and insertion of the muscles, and distinction of a joint with associated ligaments.

A refining process was carried out in dyads (i.e. SP with each junior instructor) to improve the junior instructors' prosection skills. On special occasions, the whole group was gathered, and the senior instructor emphasized critical points, e.g., identifying and exposing the radial nerve in the axillary region.

Each junior instructor completed eight preparates (a prosected body part, e.g., pelvis and thigh including pelvic and thigh musculature and neurovascular structures) and prosected two regions of a cadaver (e.g., posterior thigh and anterolateral forearm) based on 350 key points/words. A total of eight cadavers were fully prosected, and 32 preparates were prepared as part of the training program, and were later used for the prosection laboratories.

#### The workshop on principles of anatomy instruction

This workshop was conducted by two senior instructors (SP and AB) during April and May of each year, before the beginning of the prosection laboratories (Supplementary material 1: Workshop on principles in Anatomy instruction). This workshop emphasized pedagogical and didactical aspects and principles of teaching in small groups. In addition, on each day, the junior instructors practiced in dyads on a given topic, while giving and receiving feedback and reflecting on their performance. The beginning and end of each day were dedicated to sharing knowledge and preparing for the next meeting, based on the prosection laboratory syllabus.

Based on Harden and Laidlaw and Wolf et al., we added Situational Awareness (SA) as a didactical goal to this workshop [9, 65]. SA is defined as the junior anatomy instructor being aware of the learners’ focus ability and ability to hear and see demonstrations.

#### OJT

**Fig. 2:**
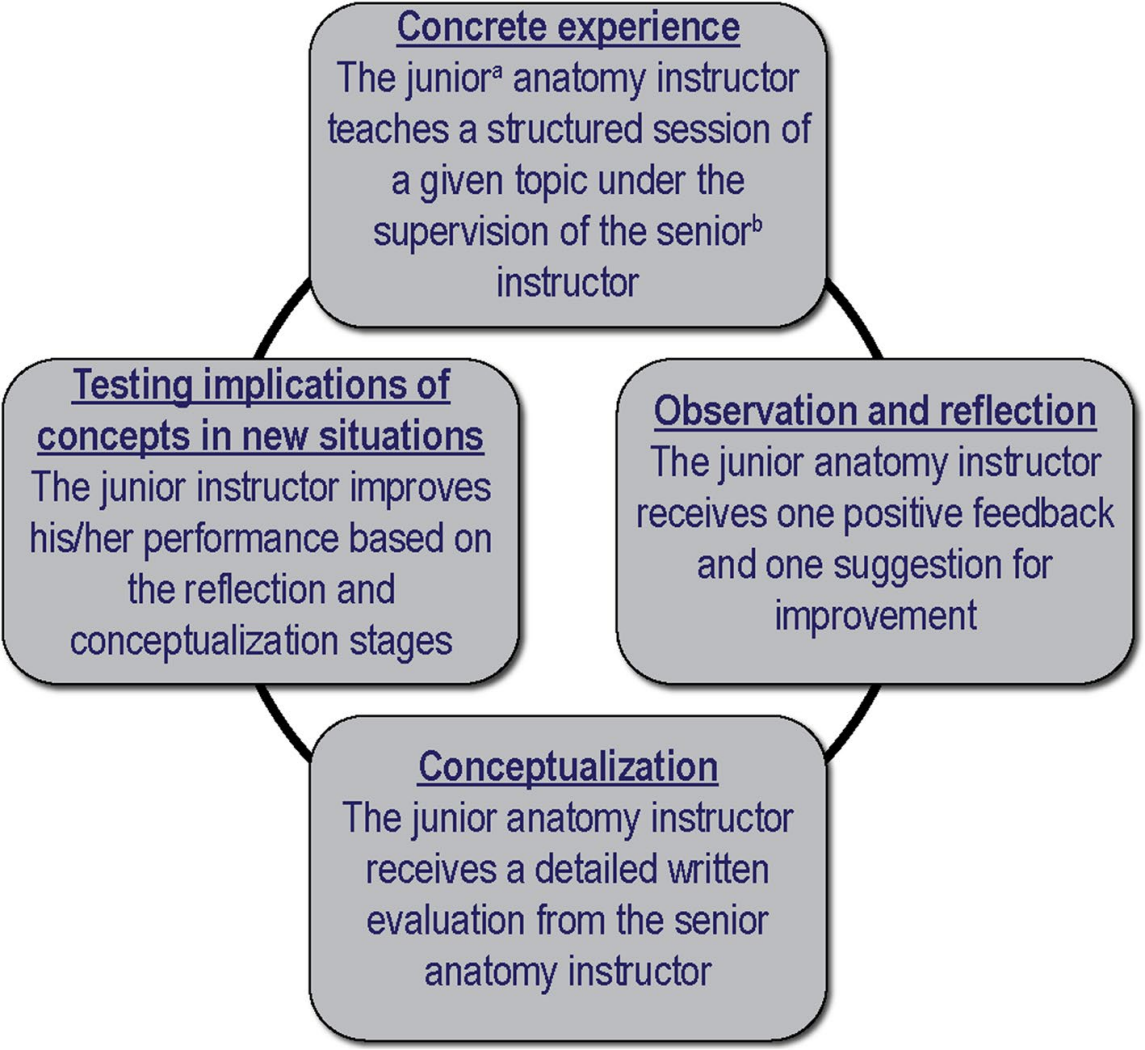
On the job training for junior anatomy instructors based on the Lewinian experiential model [63]. ^a^junior anatomy instructors 2 years of experience, ^b^senior anatomy instructors 10 years of experience

Concepts from the Lewinian experiential model (Fig. 2) were adapted to anatomy instruction and implemented as follows (May–June) [63]: every evening before the actual prosection laboratory the junior instructors simulated the material of their planned instruction in dyads. Additionally, OJT was carried out by one senior instructor (SP) during and following the prosection laboratories.

Emphasis was placed on the following [63, 64]:

- *Concrete experience and observation:* each junior instructor was observed by a senior instructor while teaching on a given topic during a prosection laboratory.
- *Observation and reflection:* after the teaching session, each junior instructor reflected on his/her performance, followed by focused feedback from the senior instructor. The feedback included one positive point for preservation and one suggestion for improvement using examples from the instructors' performance.
- *Conceptualization:* detailed written feedback was then given to each junior instructor, allowing for further clarifications if needed.
- *Testing implications of concepts in new situations:* based on the above, the junior instructors had an opportunity to improve their performance in subsequent prosection laboratories.

Each junior instructor participated in four OJT sessions, i.e. OJT was provided for each instructor during each of the prosection laboratories. During each prosection laboratory, each junior instructor gave a structured session on an anatomical region. Every session lasted 45 min, after which the groups of students alternated between the instructors. Since there were four groups of students, the junior instructors taught the same session four times. The senior instructor observed each junior instructor during one of the four sessions, and switched to the next junior instructor after the session ended. A 30-min break followed every two sessions. During the break, two junior instructors received feedback from the senior instructor comprising of one point of preservation and one suggestion for improvement. The other two junior instructors received their feedback at the end of the following two sessions. All feedback was given as a group discussion.

Written feedback was sent from the senior instructor to each of the four junior anatomy instructors. The junior instructors were encouraged to respond and ask for clarifications if and when needed.

### Prosection laboratories

Eighty-five first year PT students participated in prosection laboratories carried out by junior instructors between 2017–2018. Eighty-one PT students that participated in prosection laboratories and were taught by senior instructors between 2015–2016 served as a control group.

The prosection topics were based on clinically meaningful content for the musculoskeletal anatomy syllabus [67] and adapted to the PT curriculum, as the topics need to be relevant to the PT students’ clinical practice [3]. A list of 350 keywords of possible structures was handed out to the students in advance and served as a learning aid.

Five prosection laboratories, four hours each, took place in May–June each year. The first four laboratories were structured, whereby each junior instructor gave a teaching session on an anatomical region. The class was divided into four groups of students that rotated between the stations every 45 min, with a 30-min break after two sessions (90 min). A ratio of 10:1 between students and instructors was similar to previous years (2015–2016) when the prosection laboratories were taught by the senior instructors.

The fifth prosection laboratory served as a review session with all instructors available to answer questions.

Based on a gradual transition to student-centered learning, the students used activities and content provided by the junior instructors as part of the teaching sessions to support and facilitate the students’ learning process [66, 68].

One senior instructor (SP) supervised the junior anatomy instructors during the prosection laboratories, while the other (AB) coordinated the prosections, i.e., was available to the students’ needs. He also taught one structured session. Both senior instructors taught during the review laboratory, where the students learned independently in dyads or groups of three.

### Questionnaires

Two questionnaires were used to evaluate the educational impact of the training program, and one questionnaire was used to evaluate the junior anatomy instructors’ performance (Fig. 1) [36]. Before filling out the questionnaire, two statements were presented to the participants, one guaranteeing their anonymity and the second inviting them to volunteer for the research.

All junior instructors completed two questionnaires to self-evaluate their professional improvement following the training program and OJT. These questionnaires were developed based on Shiozawa et al., and adapted to the needs of our program [45]. The first questionnaire (Q1) was administrated at the end of the two workshops (Supplementary material 2), while the second questionnaire (Q2) was administered after the OJT process was completed (Supplementary material 3).

After completing all prosection laboratories, the students completed one questionnaire (Q3) (Supplementary material 4). This questionnaire was developed and validated by the Center for Teaching Advancement of Zefat Academic College and evaluates instruction performance, as well as interaction and atmosphere in the dissection laboratory.

All questionnaires were in the form of a Likert scale of 1–5 for questionnaires one and two, and a scale of 1–7 for the third questionnaire.

### Statistical analysis

Descriptive statistics and statistical analyses were carried out using SPSS)IBM SPSS Statistics for windows, Version 22.0, IBM Corp. Armonk NY). Statistical significance was set at α < 0.05.

Students' demography was analysed and compared between the academic years, including age, sex distribution and academic achievements (i.e. final grades in the Anatomy course and average grades of the first year). As data were not normally distributed, the Kruskal–Wallis test was used to examine differences in grades between the academic years and Mann–Whitney test used to determine differences between students taught by junior instructors and those taught by the senior ones (α < 0.05).

The reliability of all questionnaires was assessed by calculating internal consistency. The median scores were calculated for all questionnaires, and for Q3 comparisons between senior and junior instructors were carried out using the Mann–Whitney test (α < 0.05).

## Results

### Demography

A total of 164 first-year PT students participated in the study between the years 2015–2018. There were no significant differences between the academic years regarding age and first-year academic achievements (*p* = 0.925 and 0.208, respectively, Table 2). The grade in the Anatomy course in 2015 was slightly lower compared to all other years. However, the difference was significant only compared to 2017 and 2018 (*p* = 0.021, Table 2).

**Table 2:** Demography of students

Academic year	2015	2016	2017	2018	*p*
N	*n* = 43	*n* = 38	*n* = 41	*n* = 44	
Age	25.7	25.5	24.9	25.3	0.925
(SD)	(4.49)	(4.45)	(2.53)	(2.34)	
Average grades 1^st^ year	83.4	84.9	85.2	84.5	0.208
(SD)	(4.98)	(5.29)	(5.93)	(5.97)	
Anatomy course grade ^a^	80.9	83.5	85.5	85.4	*0.021*^*b*^
(SD)	(7.64)	(7.52)	(5.89)	(6.70)	
Sex Ratio	29:14	15:23	20:20	26:17	
Female: Male					

^**a**^ Kruskal Wallis test

^***b***^ Significance α < 0.05

### Reliability and validity of the questionnaires

#### Reliability

Internal consistency of all questionnaires was high (α = 0.817 for Q1 and Q2, α = 0.971 for Q3).

#### Validity

questionnaires Q1 and Q2 possess judgment-based validity, i.e. both have face and consensual validity. The panel, made up of the three senior instructors (SP, RPK and AB), agreed that all the questionnaire items were representative of the original questionnaires.

### Self-evaluation of the workshop—Q1

All junior anatomy instructors (8/8) filled out the self-evaluation questionnaire of the workshop. All didactical aspects in the workshop received a median score of 4.5 or higher on a five-point Likert scale (Supplementary material 2).

In the open comment section, the junior instructors emphasized the importance of simulations as part of the workshop. We quote:

Junior instructor 1 (2018): “Simulations were particularly important, especially before our first teaching sessions, when stage fright was high. The simulations offer additional practice in addition to the feedback and are therefore very important. The feedback enabled me to focus better during demonstrations and helped improve my instruction”.

### Self-evaluation of OJT during the prosection laboratories—Q2

All junior anatomy instructors (8/8) filled out the self-evaluation questionnaire of the OJT (Supplementary material 3). All didactical aspects of the OJT process received a median score of 4.5 or higher on a five-point Likert scale. Examples of points for preservation and suggestions for improvement are described in Table 3. The main didactical aspects were complementary to the ones taught in the workshop.

**Table 3:** Examples of the feedback given to junior instructors during OJT: a) points for preservation, and b) suggestions for improvement

**a) Points for preservation**	
**Topics from the training program**	**Examples of feedback to the junior instructor**
Combining the use of a cadaver and preparate^a^ to complete a 3-D and multilayer explanation	When explaining about the suboccipital muscles, you added a preparate and clarified the muscles' action
Providing a short clinical context when relevant	You strengthened students’ understanding by demonstrating the effect of elbow movement on the sliding motion of the long head of biceps in the bicipital groove, and related it to glenohumeral joint pathologies
Situation awareness	After the demonstration of the lumbosacral plexus, you switched places between students so everyone could see, and continued with the demonstration
**b) Suggestions for improvement**	
**Topics from the training program**	**Feedback to the junior instructors**
Presenting the topics of a session and keeping to a structured instruction	When you started your instruction, you forgot to present the main topics. This introduction will help students maintain focus
Short instruction and internalization of knowledge	Instruction should be kept to 15 min: When you explained about the different compartments of the leg, you spoke for over 25 min. You should stop after each compartment and ask the students to identify the anatomical structures
Situation awareness	You should make sure that all students can see and hear your demonstration: Failing to switch places between students during your demonstration interfered with students’ ability to see and understand the anatomical structures passing behind the medial malleolus

^a^ Preparate – a prosected body part, e.g., pelvis and thigh including pelvic and thigh musculature and neurovascular structures

### Students' evaluation of the anatomy instructors’ performance—Q3

**Fig. 3:**
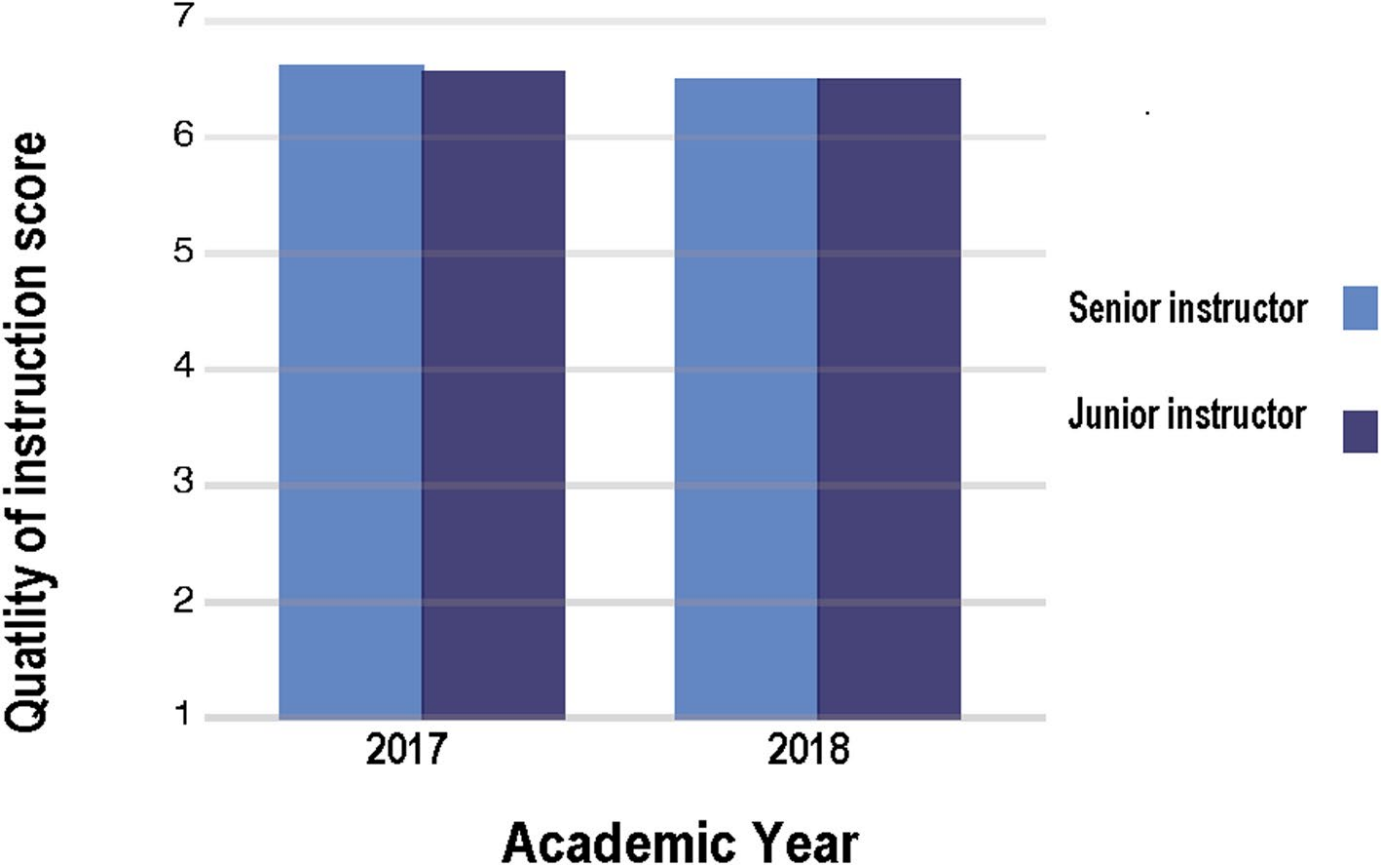
Average scores for quality of instruction for junior and senior anatomy instructors. Results of questionnaire 3 filled out by first-year PT students (*n* = 85). Possible answers were: 1 = very low, 2 = quite low, 3 = low, 4 = moderate, 5 = high, 6 = very high, 7 = extremely high. 78% percent of the students in 2017 (32/41) and 82% of the students in 2018 (36/44) filled out the questionnaire

Seventy-eight percent of students in 2017 (32/41) and eighty-two percent of students in 2018 (36/44) filled out this questionnaire (Supplementary material 4). High satisfaction rates were given to the junior and senior anatomy instructors (an average score of 6.4 or higher on a 1–7 Likert scale) (Fig. 3).

The students emphasized their understanding and satisfaction with the junior instructors in the open comments. We quote:

Student A: “The fact that the junior anatomy instructors recently graduated allowed them to focus on what was really important and relevant to our learning.”

## Discussion

The current study describes the implementation of a two-stage training program of junior anatomy instructors specifically designed for prosection laboratories. The training program consisted of two workshops and OJT, which was based on the Lewinian experiential model and embedded in the prosection laboratories [63, 64]. Additionally, simulations were included as a preparatory stage and were carried out every evening before each prosection laboratory.

The workshops followed previous publications that focused on dissection laboratories [41, 44]. Shiozawa et al., presented mini-teaching modules of short tasks and the microteaching method of short teaching sessions as key elements of professional and didactical training [41]. These short exercises offered the possibility of simulating a teaching session in a safe environment and individual reflection on one's performance, using videotape analysis. Dickman et al., taught the basics of didactics, pedagogical approaches, and teaching methods via specific modules, with emphasis on providing effective feedback as well as advanced practical skills in cadaveric dissection, without the aid of videotape recording [44]. We adopted this approach with emphasis on a more graded training process, which included preparation of the prosections by the junior instructors, simulation on their peers during the preparatory stage (i.e. the other junior instructors), followed by feedback and an opportunity to implement improvements during their real-time performance. This graded process reduces apprehension, and strengthens the integration between professional and didactical knowledge in a safe environment [69, 70]. In addition, by providing feedback to their peers, the junior instructors improved their communication skills. This important pedagogical goal, termed “dialogic communication", is considered a keystone for a student-centered approach [30, 44].

The second stage of the training program included OJT of the junior instructors, carried out during the prosection laboratories, emphasizing structured feedback, the reflection of the junior instructors on their performance, and implementation of suggestions for improvement during the following sessions.

The novelty of this study is that OJT was an integral part of the training program and corresponds with Hendry and Shiozawa et al., who raised the need for a follow-up program with formative and supervisory elements, appropriate resources allowing [41, 47]. Previous reports included weekly one-hour debriefing sessions facilitated by a core faculty member, in which the junior instructors could reflect on their personal experience, strengths and areas of improvements [36, 40].

Models for organizing and assessing junior instructors in PT programs were presented previously for NPT only [32, 33]. These reports were based on mentoring and an apprenticeship approach [36]. However, the element of individual structured supervision by a senior instructor has not been described in detail so far. Evans and Cuffe reported that the lead faculty member informally observed each NPT during each dissection laboratory, and senior instructors were available to help when any difficulty arose; however, they did not present a detailed description of the process [46].

In the current study, OJT of junior instructors included two aspects of the feedback given by the senior instructor: the first consisted of one point for preservation and one suggestion for improvement (given during the prosection laboratory, after the junior instructors reflected on their performance), the second was a detailed written feedback supported with examples. The first part enabled prompt feedback and reflection, with an immediate opportunity to implement improvement in real-time [71, 72].

This part was carried out as an open discussion and is complementary to the learning process. As was suggested by Nicol and MacFarlane-Dick, increasing discussion and reflection about criteria and standards in class promotes the trainees' performance [73]. Thus, the junior instructors learned from each others' performance and enhanced their skills by identifying performance standards [70, 73]. Therefore, in the current study, all junior instructors could improve their performance in at list one aspect based on the reflection and conceptualization stages carried out [53].

The second part followed the written feedback process described by Dickman et al., and the supervision by a senior instructor briefly described by Evans and Cuffe [44, 46]. As cameras or videotape are not allowed in the dissection room at our disposal, we could not adopt the video analysis component of the microteaching method [41].

In the current study, the senior instructor documented examples of positive performance and suggestions for improvement in writing. In addition, the junior instructors were encouraged to consult with the senior instructor in order to improve their performance [36, 40].

This slower and deeper process helped the junior instructors prepare themselves for the following laboratory, overcoming blind spots which impaired their primary performance [59, 62, 74]. Reflection and conceptualization (the third stage in the lewinian experiential model) improved the junior instructors’ ability to give a multilayered three-dimensional explanation combining the use of a prepatrate and a cadaveric prosected material [53]. For example:

Junior instructor 3 (2018): in response to the feedback I received during the first OJT session, I implemented the suggestion for improvement by adding a defleshed preparate of the wrist in order to clarify the carpal tunnel's bony boundaries. After that, I switched to the fleshed prosection of the carpal tunnel to present the different anatomical structures and how they relate to each other. I found that the combination of both preparates enhanced students' understanding, and I feel that receiving the feedback helped me improve my teaching skills.”

In this sense, we would like to emphasize the importance of providing written feedback as a key element in the junior instructors’ professional development, as it is used as part of their preparation for upcoming teaching sessions, as well as for preparing for sessions in the following academic year.

Based on the self-reported measures of the junior anatomy instructors, all didactic aspects were rated high, indicating the high educational value of the training program. This work is in line with Shiozawa et al., whereby NPT evaluated their performance before and after the training program, and an average increase of more than two points was presented for both technical and didactic aspects [41, 45]. Similar results were reported by Erie and colleagues and by Lachman et al., whereby over 90% of the NPT agreed or strongly agreed that they could effectively communicate complex material and that they were exposed to a variety of teaching techniques after obtaining teaching experience themselves in the anatomy course [36, 40].

The results of the current study showed similar academic achievements of PT students taught by junior instructors compared to those taught by senior ones and are consistent with Kinirons et al. [27]. They reported similar academic achievements of PT and occupational therapy students taught by peer teachers compared to demonstrations taught by senior faculty during dissection laboratories.

The findings from this study were further validated by the students' high evaluation rates for both junior and senior anatomy instructors. This is in agreement with Dickman et al., who reported similar evaluation rates for NPT and senior instructors, and Durán et al., who reported that 90% of students thought that the performance of NPT and professors alike was equal to a score of 8 or higher (on a 1–10 Likert scale) [31, 44]. They concluded that the quality of teaching provided by NPT is comparable to that of associate professors [31].

In the current study, PT graduates were incorporated into the prosection laboratories of a PT undergraduate program with the long-term goal of employing them within the department. During the two consecutive years of OJT, four junior instructors taught the students, one senior instructor served as their tutor and the second served as the laboratory coordinator. Therefore, six instructors were simultaneously present in each prosection laboratory, making the impression that this intervention is time and effort consuming in the short term. However, the great benefit is in the long term, i.e., starting at 2019 academic year the junior instructors conducted the prosection laboratories with only one senior instructor coordinating the laboratories. This is in line with Richardson-Hatcher et al., suggesting that structured OJT can be implemented in training programs focused on junior faculty staff development or in NP teaching programs that are part of the anatomy course in medical or health profession faculties [39].

### Limitations and future research

This study is not without limitations. First, our cohort included a small number of participants, primarily males, from a physical therapy department, limiting our results' generalizability. Second, we report on the results of OJT intervention during two consecutive academic years only. Third, we only assessed internal consistency, as it was technically impossible to administer the questionnaires again within the study period. Forth, the participation of different students according to the years may affect our findings. Lastly, we did not assess confounding factors such as self-motivation, or implicit biases that may be related to inclusion of female instructors.

Future research should involve more students and junior instructors with more senior instructors carrying out OJT including quantitative measures, implementing such programs in other health care departments, and utilizing a longer follow up periods to yield more conclusive and generalizable results. Lastly, a test–retest analysis of the questionnaires is warranted.

## Conclusions

OJT is applicable in a small-sized PT program facing a shortage of anatomy instructors and on a broader scale, adds an adaptation of the experiential learning model to the needs of a prosection laboratory. Formative and constructive feedback given to the junior instructors and simulations of teaching sessions further enhance the process.

Including junior anatomy instructors in prosection laboratories for PT students is a viable long-term solution, provided that close supervision and structured OJT is carried out.

## Supplementary Information

**Additional file 1.**

**Additional file 2.**

**Additional file 3.**

**Additional file 4.**

## Data Availability

All data generated or analysed during this study are included in this published article [and its supplementary information files].

## Abbreviations

OJT: On the Job Training
NP: Near Peer
NPT: Near peer teacher
PT: Physical Therapy
