# Supplementary material for: On the job training in the dissection room: from physical therapy graduates to junior anatomy instructors
Source: BMC Med Educ. 2022 May 10;22:354. doi: 10.1186/s12909-022-03390-y (PMC9092715; doi:10.1186/s12909-022-03390-y)
Supplement: Supplementary file 1 — Additional file 1. [file 12909_2022_3390_MOESM1_ESM.docx]

**Supplementary material/Online resource 1**

**Training program - Workshop: Principles in Anatomy instruction**

1. **How to open first lesson in the dissection room**
   1. Short debriefing by the senior instructors (AB and SP) prior to entering the dissection room regarding technical guidance of conduct in the dissection room, e.g., dress code, masks and gloves.
   2. Ethical considerations in the dissection room: addressing concerns of students facing cadavers; emotional and religious aspects (50% of our students are from traditional/religious backgrounds); importance of covering head/face and genitals of a cadaver, addressing sensitivity and respect for the dead.
   3. Orientation and identification of anatomical tissues (e.g. how to orient the students as to right/left/medial/lateral, how to differentiate a nerve from an artery/vein).
2. **Differences between teaching on a preparate and a cadaver**

Deciding when to use a preparate or a cadaver, or both: a preparate can be viewed from all directions, whereas a cadaver allows for a holistic view. Deciding when a combined use of a cadaver and preparate is warranted for a complete understanding of the anatomical region studied: e.g., when studying the ankle and foot region on the cadaver one can see the first layer of musculature and general contour, whereas supplementation with a preparate to demonstrate the deeper layers and a defleshed preparate of joints and ligaments will deepen the understanding.

1. **Teaching skills:**
   1. Principles on teaching in small groups.
   2. Identifying weak students: balancing different learning abilities during a demonstration, recognizing students' engagement level.
   3. Focused explanation: focusing on an anatomical tissue/region and avoiding elaborate and complicated explanations, as it might confuse students.
   4. Providing a short and relevant clinical context when indicated: first year PT students lack clinical context as the relevant courses are taught in subsequent years; e.g., when demonstrating on a preparate the Gleno-humeral joint, Supraspinatus tendon and Long head of biceps, a short clinical context regarding pathology of these tendons should be provided.
   5. Situational awareness: how to make sure all students see and hear the demonstration. When needed, rotating between students' positions, and repeating the explanation/demonstration. How to recognize the level of engagement and understanding of students.
   6. Verifying understanding:
      1. During demonstration, after explaining five anatomical structures the instructor proceeds with review questions to ensure all students understand the demonstration.
      2. After each teaching station, review questions are used regarding the anatomical structures.
2. **Simulation before prosection laboratories:**
   1. Preparation of teaching sessions by choosing key words (i.e. representing 15 anatomical structures) that will be taught to students. The sessions were based on the prosection laboratory syllabus.
   2. Planning each session based on the above teaching methods (i.e. section 3).
   3. Practicing in dyads on a given topic, while giving and receiving feedback and reflecting on each other’s performance.
   4. Practicing on a group (i.e. on the other junior instructors) and receiving feedback from the senior anatomy instructor (SP).
