# Supplementary material for: On the job training in the dissection room: from physical therapy graduates to junior anatomy instructors
Source: BMC Med Educ. 2022 May 10;22:354. doi: 10.1186/s12909-022-03390-y (PMC9092715; doi:10.1186/s12909-022-03390-y)
Supplement: Supplementary file 2 — Additional file 2. [file 12909_2022_3390_MOESM2_ESM.docx]

**Supplementary material/On line resource 2**

**Questionnaire 1 – Self-evaluation of the workshops ^a^**

| On a scale of 1-5 please rate to what extent the training program impacted the following skills ^a^ | 2017^b^  (n=4)  Median | 2018^b^  (n=4)  Median |
| --- | --- | --- |
| Improved my ability to describe an anatomical structure | 4.5 | 4.5 |
| Improved my ability to describe three-dimensional structures | 5.0 | 4.5 |
| Improved my instruction abilities in front of a group | 4.5 | 4.5 |
| Improved my recognition of essence | 4.5 | 5.0 |
| Improved my ability to maintain focus | 4.5 | 4.5 |
| Enhanced my knowledge in anatomy | 5 | 4.5 |
| Enhanced my clinical knowledge | 5 | 4.5 |

^a^ Possible answers: 1=strongly disagree, 2= disagree, 3=neutral 4=agree, 5=strongly agree.

^b^ A median score shown for all junior instructors.
