# Supplementary material for: On the job training in the dissection room: from physical therapy graduates to junior anatomy instructors
Source: BMC Med Educ. 2022 May 10;22:354. doi: 10.1186/s12909-022-03390-y (PMC9092715; doi:10.1186/s12909-022-03390-y)
Supplement: Supplementary file 4 — Additional file 4. [file 12909_2022_3390_MOESM4_ESM.docx]

**Supplementary material/On line resource 4**

**Questionnaire 3 – Evaluation rate of instruction ^a^**

| On a scale of 1-7 please rate the following ^b^ | Junior instructors  2017 ^c^  Mean (SD) | Senior instructors  2017 ^c^  Mean (SD) | Junior instructors  2018 ^c^  Mean (SD) | Senior instructors  2018 ^c^  Mean (SD) |
| --- | --- | --- | --- | --- |
| The goals of the dissection laboratory were clear | 6.66  (0.66) | 6.67  (0.63) | 6.41  (0.98) | 6.24  (1.23) |
| The dissection laboratory stimulated me to independent thinking | 6.58  (0.76) | 6.58  (0.79) | 6.49  (0.88) | 6.44  (1.0) |
| The dissection laboratory provided me tools that improved my understanding in anatomy | 6.61  (0.69) | 6.82  (0.44) | 6.33  (1.26) | 6.42  (0.94) |
| General estimation of the dissection laboratory | 6.63  (0.70) | 6.76  (0.49) | 6.48  (0.96) | 6.37  (1.02) |
| The instructors' explanations were clear | 6.48  (0.85) | 6.69  (0.70) | 6.21  (1.17) | 6.34  (1.12) |
| The instructor taught in a well-organized manner | 6.60  (0.63) | 6.54  (0.91) | 6.30  (0.95) | 6.25  (1.21) |
| General estimation of the tutoring quality of the anatomy instructor | 6.58  (0.66) | 6.68  (0.59) | 6.27  (1.06) | 6.39  (1. 04) |
| The instructor induced a respectable learning atmosphere during the dissection laboratory | 6.71  (0.58) | 6.76  (0.55) | 6.30  (1.13) | 6.39  (1. 04) |
| I felt that the anatomy instructor was concerned and obligated to our success | 6.73  (0.54) | 6.70  (0.57) | 6.35  (1.06) | 6.39  (1. 04) |
| The anatomy instructor was attentive and available to the students beyond the course hours | 6.53  (0.81) | 6.59  (0.78) | 6.23  (1.10) | 6.39  (1.04) |
| General estimation of the interaction between the instructor and the students | 6.68  (0.62) | 6.73  (0.55) | 6.47  (0.80) | 6.44  (1.0) |

^a^ Developed and validated by the unit for promoting teaching in Zefat Academic College. Mean scores presented for the three junior and two senior instuctors.
^b^ Possible answers: 1=strongly disagree, 2= disagree, 3=somewhat disagree, 4=neutral, 5=somewhat agree, 6=agree, 7= strongly agree.

^c^ Junior instructor years teaching experience, n=4 each year; senior instructor 10 years teaching experience, n=2 each year
